# Magnetic Chitosan for the Removal of Sulfamethoxazole from Tertiary Wastewaters

**DOI:** 10.3390/nano14050406

**Published:** 2024-02-23

**Authors:** Domenico Pirozzi, Alessandro Latte, Abu Yousuf, Francesco De Mastro, Gennaro Brunetti, Andrea EL Hassanin, Filomena Sannino

**Affiliations:** 1 Department of Chemical Engineering, Materials and Industrial Production (DICMaPI), Laboratory of Biochemical Engineering, University of Naples “Federico II”, Piazzale Tecchio, 80, 80125 Naples, Italy; domenico.pirozzi@unina.it (D.P.); alessandro.latte@gmail.com (A.L.); andrea.elhassanin@unina.it (A.E.H.); 2 Department of Aerospace and Mechanical Engineering, University of Oklahoma, Norman, OK 73069, USA; ayousufcep@yahoo.com; 3 Department of Soil, Plant, and Food Sciences, University of Bari Aldo Moro, 70126 Bari, Italy; francesco.demastro@uniba.it (F.D.M.); gennaro.brunetti@uniba.it (G.B.); 4 Department of Agricultural Sciences, University of Naples “Federico II”, Via Università 100, 80055 Naples, Italy

**Keywords:** magnetic chitosan, sulfamethoxazole, wastewater, adsorption

## Abstract

Magnetic chitosan nanoparticles, synthesized by in situ precipitation, have been used as adsorbents to remove sulfamethoxazole (SMX), a sulfonamide antibiotic dangerous due to its capacity to enter ecosystems. The adsorption of SMX has been carried out in the presence of tertiary wastewaters from a depuration plant to obtain more realistic results. The effect of pH on the adsorption capacity significantly changed when carrying out the experiments in the presence of wastewater. This change has been explained while taking into account the charge properties of both the antibiotic and the magnetic chitosan. The composition of wastewaters has been characterized and discussed as regards its effect on the adsorption capacity of the magnetic chitosan. The models of Elovich and Freundlich have been selected to describe the adsorption kinetics and the adsorption isotherms, respectively. The analysis of these models has suggested that the adsorption mechanism is based on strong chemical interactions between the SMX and the magnetic chitosan, leading to the formation of an SMX multilayer.

## 1. Introduction

Sulfamethoxazole (SMX) is an antibiotic belonging to the sulfonamide class, commonly used in combination with trimethoprim to treat bacterial infections [1]. Its antibiotic effect is associated with the inhibition of the dihydropteroate synthase, which is a precursor of folic acid and is consequently associated with bacterial growth [1].

In recent years, the widespread use of SMX has led to its release in large amounts, and eventually to its contact with bacterial communities and ecosystems. The environmental impact of SMX has become a concern as it can enter water bodies through the discharge of treated or untreated sewage, as well as through runoff from agricultural areas where it may be used in livestock [2].

SMX has been shown to have adverse effects on aquatic organisms, as it can interfere with the growth and reproduction of algae, as well as impact the physiology and behavior of fish and other aquatic organisms. Its bioaccumulation in aquatic organisms may lead to higher concentrations in organisms higher up the food chain.

Moreover, the antibiotic nature of SMX can contribute to the development of antibiotic resistance in environmental bacteria. As an antibiotic, it can disrupt microbial communities in soils and water bodies. This disruption may have cascading effects on ecosystem functions and services.

The concerns associated with SMX pollution are also due to its significant persistence in the environment. It can resist degradation processes in water, soil, and sediments for extended periods. Transformation products can also be formed through chemical and biological processes, leading to the generation of metabolites that may have different environmental fates and effects.

Consequently, the presence of SMX in the environment has led to increased attention from regulatory agencies. Efforts are being made to monitor and regulate the release of pharmaceuticals into the environment. Efforts to mitigate the environmental impact of pharmaceuticals often involve improved wastewater treatment, proper disposal practices, and the development of alternative medications with reduced environmental persistence.

Though different methods have been developed to remove antibiotics from wastewaters, such as advanced oxidation processes, coagulation, sedimentation, filtration, membrane technologies, and biological treatments [3,4,5,6,7], none of these approaches have given fully satisfactory results. As a matter of fact, some of these methods require additional chemicals or high amounts of energy during the process and generate waste and byproducts that have to be disposed of in subsequent steps. Reverse osmosis, ion exchange, and advanced oxidation processes, though being more attractive, do not seem to be economically feasible because of their relatively high investment and operational cost [8].

Adsorption is considered an excellent process for treating wastewater containing a low concentration of antibiotics, as it exhibits the aforementioned drawbacks to a significantly lower extent than the other processes [9,10,11,12,13]. Adsorption is a widely used and effective method for the removal of emerging contaminants from wastewater, as it is effective for removing different classes of materials: organic pollutants such as dyes, pesticides, and pharmaceuticals; heavy metals; and nutrients like phosphorus and nitrogen, potentially leading to eutrophication. The main advantages offered by adsorption are the high removal efficiency with a wide range of pollutants. In addition, different types of adsorbents can be tailored to specific contaminants, making adsorption a versatile technology, and many adsorbents can be regenerated and reused, reducing operational costs. Adsorption can be part of integrated wastewater treatment systems, complementing other processes such as biological treatment and membrane filtration [13]. Yet, some bottlenecks still limit a wider application of adsorption processes, such as the cost of adsorbents and their regeneration, the competition for adsorption sites in the presence of complex mixtures, and the complex transitioning of laboratory-scale results to large-scale applications. Another important factor still limiting the development of the adsorption processes stems from the need to separate and regenerate the adsorbent, though research activities are ongoing to solve this problem by different approaches, such as the flotation [14] of magnetic separation.

In this research, a composite material containing chitosan as the adsorbent has been used for the remediation of wastewater obtained from a tertiary treatment in a depuration plant. Chitosan is a linear polysaccharide made of N-acetyl-D-glucosamine and D-glucosamine units, increasingly used in several industrial applications due to its biocompatibility, biodegradability, non-toxicity, and chemical inertness [15]. Furthermore, chitosan is attracting growing interest as a sorbent material as the hydroxyl groups (−OH) and primary amine groups (−NH_2_) on its surface. These functional groups allow the reaction of chitosan with other compounds and, consequently, the synthesis of new composite materials with favorable properties [16]. Thanks to the high reactivity of chitosan, it is also possible to improve its adsorption capacity through the increase in its surface area and its higher affinity towards many pollutants potentially adsorbed. Interaction with Fe_3_O_4_ also enables the production of magnetic chitosan, allowing for the easier separation of the spent adsorbent from the aqueous phase [17].

In order to provide more useful results and indications, we have used SMX solution in tertiary wastewater obtained from a depuration plant fed with both urban and non-urban wastewater. As a matter of fact, the adsorption of a specific pollutant can be influenced by the presence of other components in wastewater. When a pollutant is inserted in a complex matrix like wastewater, its adsorption behavior may be affected by different factors. First of all, various contaminants may compete for available adsorption sites on the adsorbent material, potentially reducing the adsorption efficiency for the target pollutant. In addition, the presence of dissolved solids in wastewater can influence the surface chemistry of both the adsorbent and the pollutant. Ionic strength, pH, and the nature of dissolved salts can affect the electrostatic interactions and surface charge, impacting adsorption behavior.

To address these complexities, the adsorption ability of the magnetic nanocomposite in the removal of SMX from wastewaters has characterized the investigation of the role played by the main process parameters: pH, time, and the initial concentration of adsorbate.

## 2. Materials and Methods

### 2.1. Wastewaters

Wastewater was obtained from the depuration plant of Noci (Bari, Italy), fed with urban and non-urban wastewater from the province of Bari. Wastewater was preliminarily subject to a screening treatment to remove coarse materials (plastics, stones, paper, etc.). The subsequent stage aimed for the separation of sand, oils, and fats. The mechanical treatments ended with the treatment of wastewater in a primary aeration tank, where activated sludges were used to eliminate the dissolved pollutants. The sludges were then separated from the aerated mixture in the sedimentation tank and partially recirculated in the aeration tank. The water leaving the sedimentation tank was subjected to tertiary treatments to reduce the load of nutrient elements and recalcitrant organic molecules. The water purified in this way can be used in agriculture. All physicochemical parameters were analyzed according to the Standard Methods for the Examination of Water and Wastewater [18], while the quantification of pharmaceuticals in treated wastewater was evaluated using previously established analytical settings [19]. A detailed composition of the tertiary wastewater is reported in Table 1 and Table 2.

### 2.2. Preparation and Characterization of Magnetic Chitosan

Chitosan powder (medium molecular weight, deacetylation degree > 75%) was obtained from Merck KGaA (Darmstadt, Germany). In a typical test, 4.938 g of chitosan, 1.662 g of ferric chloride hexahydrate, and 0.846 g of ferrous sulphate heptahydrate were dissolved in 120 mL of ultrapure water. The mixture was stirred at room temperature and stirred (250 rpm) for 3 h. Subsequently, 0.064 mL of Tween 80 was added dropwise. The mixture was then stirred at 70 °C and stirred (250 rpm) for 2 h. The pH of the mixture was brought to a value of 12.0 by the dropwise addition of 3 M NaOH. The magnetic particles were then removed with a magnet and subjected to washing with water and ethanol. Finally, the magnetic particles were dried at 100 °C for 8 h and then at 250 °C for 30 min.

The chemical structure of magnetic chitosan before and after adsorption with SMX, at a 50 μmol/L initial concentration, at pH 3.0 or pH 7.0, and in the absence or in the presence of tertiary wastewaters, respectively, was characterized by Fourier Transform Infrared Spectroscopy (FTIR). A Jasco FT–IR 430 spectrophotometer (Jasco Europe, Cremella, Italy) was used to record the spectra. The analysis was carried out using powders mixed with KBr in a 1:100 weight ratio and then pressed under vacuum into a disk. The samples were scanned from 400 cm^−1^ to 4000 cm^−1^.

The point of zero charge (pH_PZC_) was measured following the method of [20]. SEM-EDS analyses were carried out using a Hitachi TM3000 tabletop SEM, equipped with a 15 kV electron beam and an Oxford Instruments SWIFTED3000 EDS probe. To perform the analysis, the samples (magnetic chitosan before and after SMX adsorption) were deposited on conductive carbon tape placed on a pin stub. Afterwards, the stubs were sputtered with gold by means of a Quorum Technologies K650X sputter coater machine (current: 70 mA; sputtering time: 2.5 min; vacuum: 1 × 10^−2^ mbar). SEM images were then acquired at a magnification of 500× in order to show the particles’ morphologies and to detect any variation in the latter as a function of their use and handling. EDS spectra were acquired on the same samples, but with a magnification of 100× and prior to the sputtering to avoid any signal suppression. The spectra were acquired by means of the Aztec Energy^®^ software (version 2.1) used to work with the SEM-EDS system, and data were collected for a time of 5 min for each sample.

The specific surface area and pore volume were measured following the BET and BJH methods. In this view, a Quantachrome Autorsorb 1c (Boynton Beach, FL, USA) was used to carry out the nitrogen physiadsorption. The hydrodynamic diameter of the nanoparticles was determined by photon correlation spectroscopy using an N5 Submicron Particle Size Analyzer (Beckman-Coulter, Brea, CA, USA).

The magnetic properties of nanoparticles were characterized using a vibrating sample magnetometer (Microsense Model 10).

### 2.3. Adsorption Experiments

In a typical adsorption test, 126.64 mg of SMX was dissolved in 1000 mL of ultrapure water or tertiary wastewater (500 μM/L final concentration). The resulting solution was kept refrigerated at a 4 °C temperature and used to prepare all solutions tested within this work. Batch experiments of SMX removal from water by adsorption were performed. Aqueous solutions of SMX were put into contact with 2 mg of nanocomposites in glass vials with Teflon caps at 25 °C; the vessels were continuously stirred in an orbital shaker at a speed of 400 rpm. At fixed times, 2 mg of magnetic chitosan was separated using an external magnet (VA03, UNIDISP s.r.l., Italy), and the liquid was analyzed to evaluate SMX concentration, obtained by using HPLC analysis. The amount of SMX adsorbed was calculated as the difference between its initial and final concentration in solution. Blanks of SMX in ultrapure water were analyzed in order to evaluate SMX stability and sorption on vials. The following experimental factors were evaluated:

(a) Effect of pH: Here, 2 mg of adsorbent was put into contact with 20 mL of 40 μmol/L SMX solution at a solid/liquid ratio (S/L) = 1/10,000, g/g for 24 h (this time was shown to be sufficient to attain an equilibrium condition). The pH of this solution varied between 3.0 and 8.0.
(b) Adsorption kinetics: Kinetic studies were performed by putting 2 mg of adsorbent into contact with 20 mL of SMX solution at two different concentrations, 25 and 50 μmol/L, respectively, at a solid/liquid ratio S/L = 1/10,000, at pH 3.0. The suspensions were stirred for a fixed time (5, 10, 15, 30, 60, 120, 240, 480, and 1440 min) and successively subjected to the separation procedure described above.
(c) Sorption isotherm: Here, 2 mg of adsorbent was put into contact with 20 mL of solutions having SMX concentrations of up to 250 μmol/L at S/L = 1/10,000, T = 25 °C, and pH = 3.0, for 24 h.

### 2.4. Analytical Determination

The SMX concentration in solution was measured with an Agilent 1200 Series HPLC apparatus (Wilmington U.S.), equipped with a DAD and ChemStation Agilent Software. A Macharey-Nagel Nucleosil 100–5C18 column (stainless steel 250 × 4 mm) was utilized. The isocratic mobile phase, pumped at 1.0 mL/min flow, was a binary mixture of acetonitrile with water acidified to pH 3.0 by formic acid 0.1% *v*/*v*. The ratio used was 30:70 acetonitrile/water. The detector was set at 265 nm. The injection volume was 20μL. The quantitative determination was performed using a calibration curve. The following concentration ranges were adopted: 12.5–250 μmol/L.

### 2.5. Statistical Analysis

All data are presented as mean ± standard deviation (n = 3). SPSS program (version 20) was used for the analysis, and the statistical significance (*p* < 0.05) was determined using Student’s *t*-test.

## 3. Results

### 3.1. Characterization of Magnetic Chitosan

**Figure 1:**
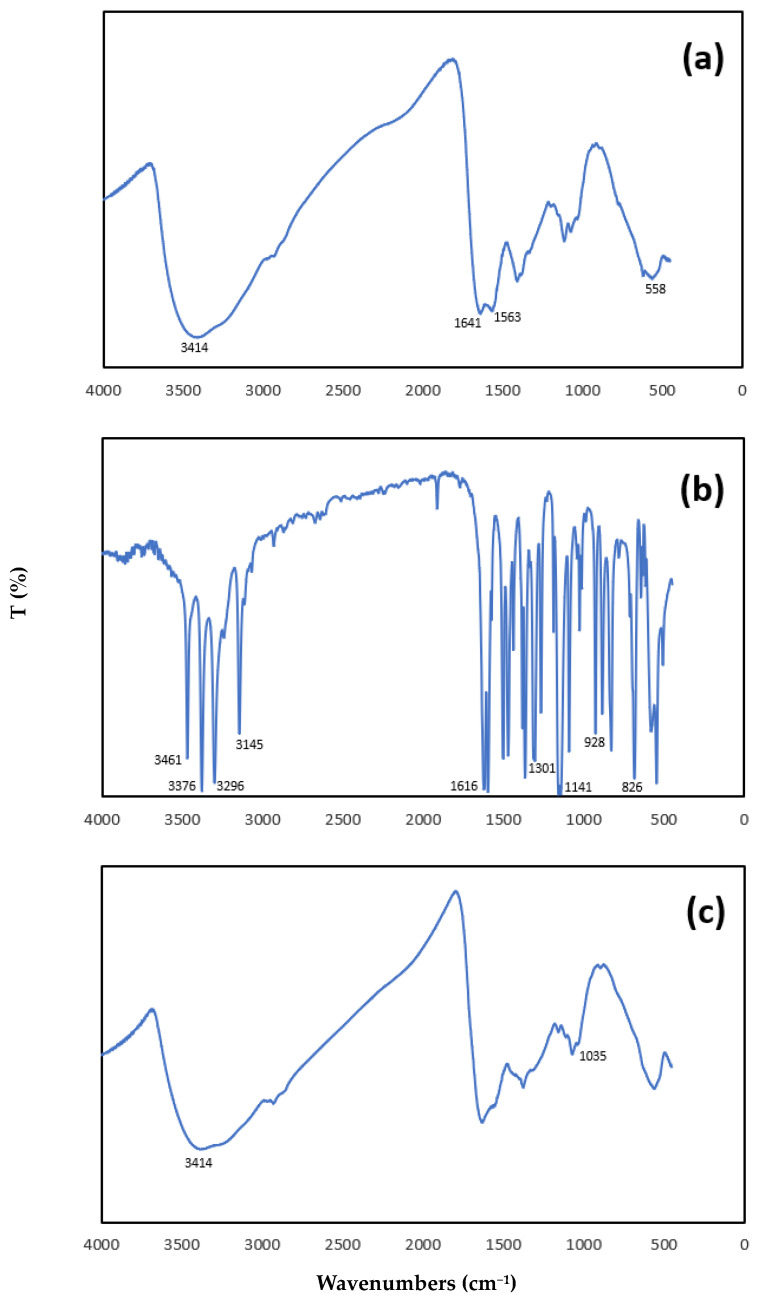
FTIR spectra: (**a**) magnetic chitosan, (**b**) SMX, (**c**) magnetic chitosan after adsorption with SMX solution at 50 μmol/L initial concentration, pH 3.0 for 24 h at 25 °C.

In order to ascertain the effective adsorption of SMX on the magnetic chitosan, we compared the FTIR spectra of adsorbate with the spectra of both the fresh and the exhausted adsorbent (Figure 1a–c). The results obtained for the magnetic chitosan sample after SMX adsorption was independent of the presence of tertiary wastewater.

In the spectrum of the magnetic chitosan (Figure 1a), we identified the typical absorption bands: the strong and broad band at 3414 cm^−1^ can be assigned to the stretching mode of the O-H and N-H bond; the band at 1641 cm^−1^ can be assigned to the stretching mode of C=O bonds in acetamido groups (amide I band); and the absorption band at 1563 cm^−1^ can be attributed to N–H bending vibration.

The sharp peak appeared at 558 cm^−1^ related to the Fe–O group, indicating that chitosan was successfully coated to the magnetic nanoparticles of iron oxides.

The characteristic bands in the FTIR spectrum of SMX (Figure 1b) were as follows:

N-H stretching vibrations at 3461 and 3376 cm^−1^ due to N-H stretching vibrations of NH2, at 3296 cm^−1^ to NH; C-H stretching and deformation modes of the phenyl ring at 3145 and 826 cm^−1^, respectively, and, at 1616 cm^−1^, a combination mode of NH_2_ and the isoxazole ring. Finally, sulphon vibrations at 1301 and 1141 cm^−1^ were detected [21].

After the adsorption of SMX onto magnetic chitosan (Figure 1c), the most significant changes were the following: a reduction in the band at 3414 cm^−1^, and a shift in the vibration of the S-N bond from 928 cm^−1^ for SMX (see Figure 1b) to 1035 cm^−1^ for magnetic chitosan after adsorption.

A consistent variation in the band at 1641 cm^−1^ and the loss of the band at 1563 cm^−1^ were observed after adsorption (Figure 1c), confirming that -NH_2_ participated in the adsorption.

**Figure 2:**
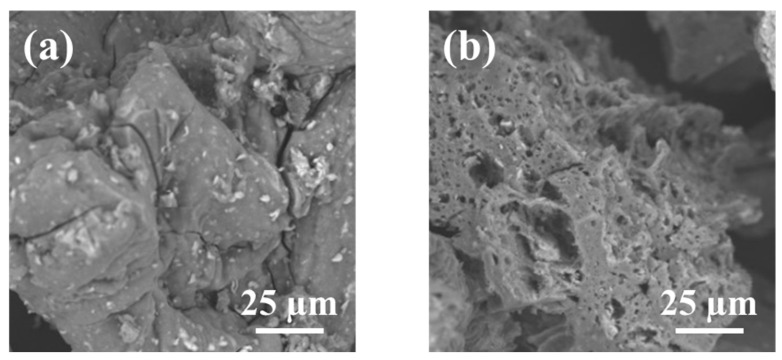
SEM images of magnetic chitosan (**a**) before and (**b**) after adsorption of SMX.

The SEM image of the magnetic chitosan in Figure 2a shows a rough surface partially covered by nanoparticles of iron oxides, while other smaller ones were embedded into the magnetic chitosan composite (Figure 2a). The Fe_3_O_4_ nanoparticles were likely immobilized on the surface through interactions with the amino and hydroxyl groups of the chitosan surface. As a result of the adsorption process, the morphology of the adsorbent changed significantly, showing a more intense porosity (Figure 2b). The results of EDS microanalysis, shown in Table 3, evidently indicate the amount of Fe on the magnetized surface of chitosan as expected and previously reported [22], and an increase in nitrogen from 9.2 to 9.7%.

In Table 4, the morphological and magnetic characteristics of the magnetic chitosan used in this study are compared with those pertaining to similar nanoparticles obtained by in situ precipitation in previous studies. The specific surface and the pore-size range obtained with the magnetic chitosan used in this study were 51.3 m^2^/g and 7.23–18.0 nm, respectively. These values are in substantial agreement with previous studies [23,24,25,26], taking into account the fact that the values of the morphological parameters reported in Table 2 are weighted averages between the characteristics of pure chitosan and pure magnetite and are consequently a function of the chitosan/magnetite ratio. In all the studies considered, the pore-size range is typical of mesopores. The value of the hydrodynamic diameter, 16 nm, is higher than those observed in previous studies [24,25,26], possibly due to a partial aggregation of the nanoparticles.

The magnetization curves of the nanoparticles used in this study (Figure S1) indicated superparamagnetic behavior, with magnetic saturation of 36.6 emu/g. This is in agreement with similar studies [23,25,26], where the magnetic saturation is, in all cases, in the range of 34–49 emu/g.

### 3.2. Effect of the pH

#### 3.2.1. Effect of the pH in the Absence of Wastewater

**Figure 3:**
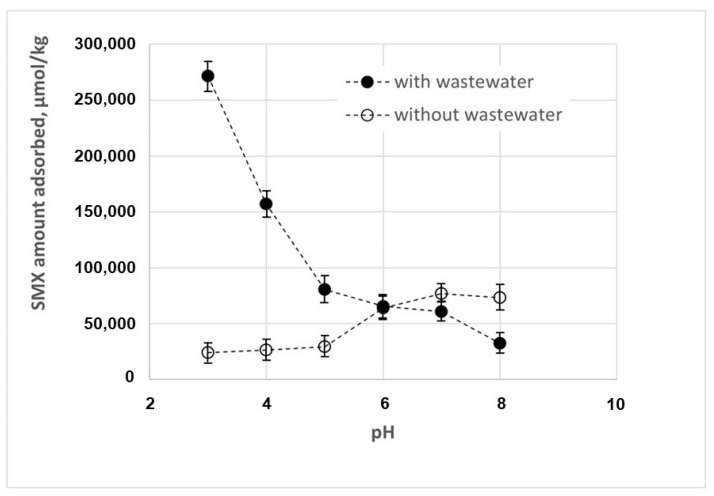
Effect of pH of SMX adsorption in the presence and in the absence of wastewater.

**Figure 4:**
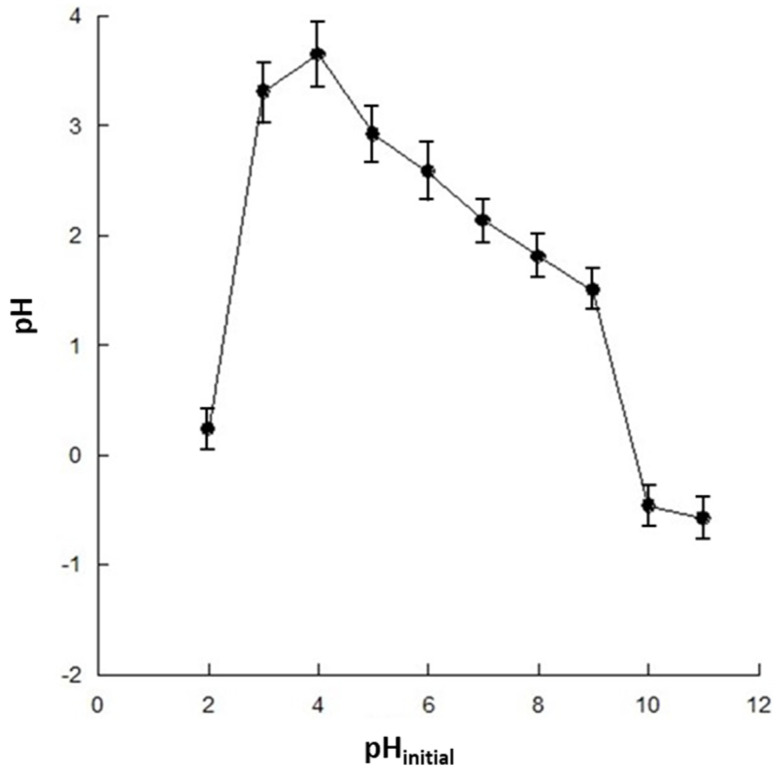
Zero-point charge of the magnetic chitosan.

The effect of pH on the SMX removal is described in Figure 3. The experimental data indicate that SMX removal by adsorption increases at higher pH values. This result can be attributed to the properties of both the antibiotic and the chitosan adsorbent. As a matter of fact, the charge of chitosan is pH-dependent, mainly due to the presence of amino groups on the glucosamine units in its structure, which can be protonated or deprotonated depending on the pH of the solution. As a matter of fact, the experimental data in Figure 4 show that chitosan is positively charged at a lower pH, whereas it has a neutral or slightly negative charge at a higher pH, with a zero-charge point of about 9.9.

This behavior can be explained by observing that chitosan is a linear polysaccharide composed of randomly distributed β-(1-4)-linked D-glucosamine and N-acetyl-D-glucosamine units that can take on different structures, shown in Figure S2, as the pH changes. The amino group of the glucosamine unit is positively charged at pH < 4.5, as shown in Figure S3. As the pH increases, the amino groups are progressively protonated, to become neutrally charged at pH > 8.5. The pKa of the amino groups in chitosan is about 6.4.

On the other hand, the chemical structure of SMX includes functional groups such as amine (NH_2_) and sulfonamide (SO_2_NH_2_) which, on the basis of their protonation state, generate three different forms of SMX, namely, negatively, neutrally, and positively charged, as shown in Figure S4. Figure S5 describes the distribution of the structures of SMX at various pH values.

From the speciation diagrams in Figures S3–S5, it can be observed that, under acidic conditions, adsorption is made difficult as both chitosan and SMX are positively charged, which results in electrostatic repulsion between the adsorbent and adsorbate.

**Figure 5:**
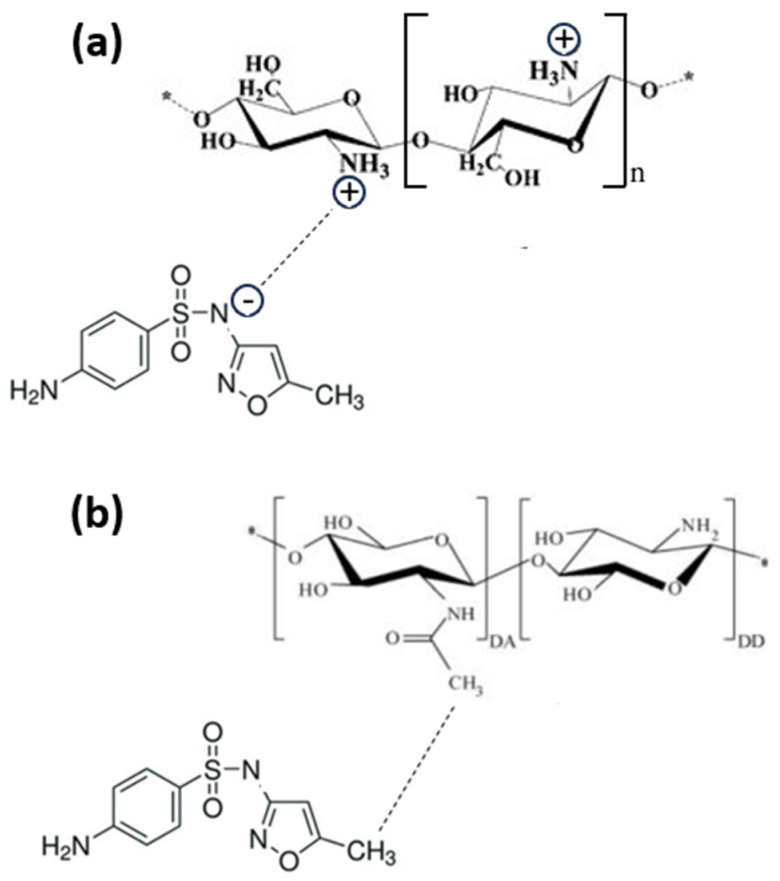
Sulfamethoxazole interaction with partially deacetylated chitosan via hydrophobic interactions (**a**) and with an amine group on chitosan surface via hydrogen bonding (**b**).

When increasing the pH values, the adsorption increases due to two competing adsorption mechanisms that may come into play, as follows:

- In the range of 4.5–8.0, both the chitosan and the SMX are partially under their neutrally charged forms, promoting nonionic interactions such as Van der Waals or hydrophobic interactions between chitosan and SMX. It is worth noting that, according to the supplier, the degree of deacetylation of chitosan is >75%. Consequently, chitosan chains contain a significant fraction of non-deacetylated units that will enhance the hydrophobic interaction, as described in Figure 5a.
- In the range of 4.0–9.5, chitosan is partially in its positively charged form and SMX is partially in its negatively charged form, and the adsorption is enhanced due to the electrostatic attractive forces. This mechanism is described in Figure 5b.

#### 3.2.2. Effect of the pH in the Presence of Wastewater

Figure 3 also shows that the effect of pH on SMX adsorption is inverted in the presence of wastewater, with a higher adsorption efficiency at lower pH values. This result is of particular importance, as wastewater can exhibit a wide range of pH values, because industrial discharges can significantly affect the pH of wastewater. Moreover, the pH level of wastewater may significantly affect the efficiency of wastewater treatments, due to its potential impact on the solubility of various compounds in wastewater, on the form of ammonia (ammonium or free ammonia), as well as on biological processes, in that the microorganisms involved typically thrive in specific pH ranges.

In order to explain the change in pH’s effect on adsorption efficiency observed in the presence of wastewater, we have characterized the physico-chemical composition of tertiary wastewaters, as shown in Table 1. On the basis of these data, it can be hypothesized that the change in the pH’s effects are mainly due to the ions contained in the wastewater. In particular, the reduced adsorption capacity observed under alkaline conditions can be explained by a lower affinity between the adsorbent and the SMX. As a matter of fact, the presence of negatively charged ions, in particular phosphate (3.31 mg/L), nitrite (0.05 mg N/L), and nitrate (0.05 mg N/L), promotes their interaction with chitosan through electrostatic interactions and other possible complexation reactions, modifying the surface charge of the chitosan and eventually its adsorption capacity. On the other hand, positively charged ions, such as ammonium (0.05 mg N/L), compete with the positively charged chitosan for interactions with the negatively charged SMX, hindering the adsorption of the latter. It has been already observed in previous studies [21,28,29] that, if the electrostatic forces between the adsorbate and the adsorbent are attractive, ions contained in the wastewater, increasing the overall ionic strength of the solution, reduce the adsorption capacity. These ions may also alter the conductivity of the solution, and eventually the mobility of ions.

Obviously, the opposite effect can be observed under acidic conditions: negative ions such as phosphate, nitrite, and nitrate may interact with chitosan through electrostatic interactions and other possible complexation reactions, reducing its positive charge. Similarly, these negative ions may interact with the positively charged SMX. As a consequence, the electrostatic repulsion between adsorbent and adsorbate is significantly reduced, promoting nonionic interactions such as Van der Waals or hydrophobic interactions, and consequently increasing the adsorption of SMX on chitosan.

Furthermore, we have analyzed the main pharmaceutical pollutants contained in the tertiary wastewater, as shown in Table 2. As a matter of fact, the presence of pharmaceuticals in wastewater has become a growing environmental concern due to their potential impacts on aquatic ecosystems and human health. Significant amounts of emerging contaminants have been detected, such as carbamazepine, diclofenac, olmesartan, and telmisartan [30].

It is worth observing that the net charge of some of these pollutant molecules can be affected by changes in the pH of the medium. For instance, diclofenac keeps its electrically neutral form under acidic conditions, whereas its anionic form is progressively formed when the pH increases, as the pKa is about 4.0 [31]. Consequently, under alkaline conditions, the negatively charged diclofenac may interact with the positively charged molecules of chitosan through electrostatic interactions, thus contributing to the reduced adsorption capacity observed in the presence of tertiary wastewaters (see Figure 3). Another example is carbamazepine, which keeps its electrically neutral form at neutral pH, whereas its cationic form is progressively formed when the pH is lower than 4.0 [32].

### 3.3. Adsorption Kinetics

**Figure 6:**
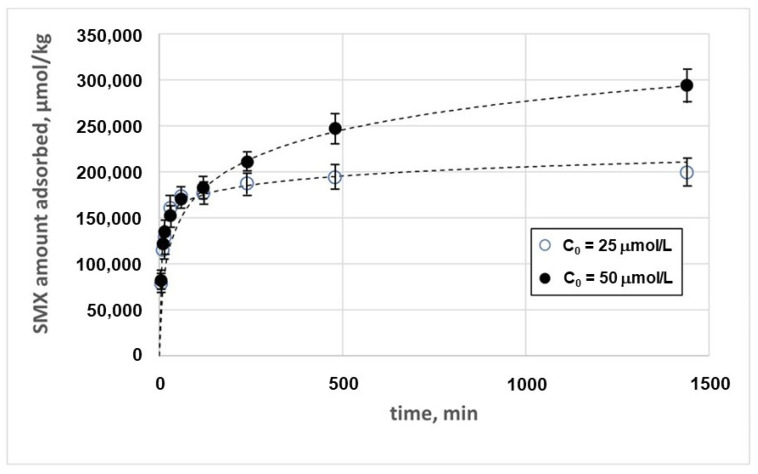
Adsorption kinetics of SMX on the magnetic chitosan in the presence of tertiary wastewater.

The kinetics of adsorption of SMX on chitosan at pH 3.0, measured by adopting two different initial concentrations of SMX, is described in Figure 6.

Different models were adopted to describe the experimental data, as described in Paragraph S1. The best results were obtained with the Elovich model as follows [33]:

(1) $$q_{t}=\frac{1}{\beta }ln\left(1+\alpha \cdot \beta \cdot t\right)$$

where *q*_e_ (μmol·kg^−1^) is the amount of SMX adsorbed per unit mass of adsorbent (adsorption capacity), *α* (μmol·kg^−1^·min^−1^) is the Elovich initial adsorption rate, *β* (kg·μmol^−1^) is the desorption constant, and *t* (min) is the time. The estimates of the parameters and the correlation coefficient obtained with the Elovich model are reported in Table 5. The results obtained with the remaining models are reported in Table S1.

The Elovich model is usually suitable for systems where chemisorption plays a significant role, suggesting that the adsorption process studied involves strong chemical interactions between the SMX and the magnetic chitosan. This model is based on the hypothesis that the progressive decrease in the adsorption rate is due to the saturation of adsorption sites and the eventual multilayer mechanism. The parameter *β*, which is related to the desorption process, is in all cases much lower than *α*, suggesting that the interaction between adsorbent and adsorbate is relatively strong and that desorption is less prominent.

### 3.4. Adsorption Isotherm

**Figure 7:**
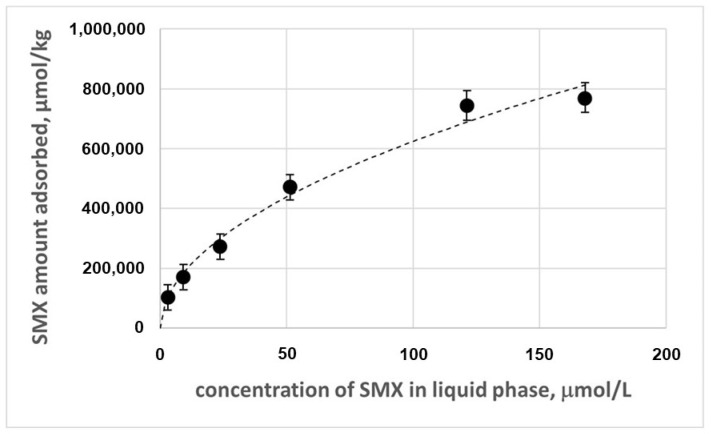
Adsorption isotherm of SMX on the magnetic chitosan in the presence of tertiary wastewater.

The adsorption isotherms corresponding to the adsorption of SMX on the magnetic chitosan are reported in Figure 7.

Different models were adopted to describe the experimental data (Table S2). The best results were obtained with the Freundlich model as follows [34]:

(2) $$q_{e}=K_{F}\cdot C_{e}^{1/n}$$

where *q*_e_ (μmol·kg^−1^) is the amount of SMX adsorbed per unit mass of adsorbent (adsorption capacity), *C*_e_ (μmol·L^−1^) is the equilibrium concentration of SMX in the solution, *K_F_* (μmol^(*n*−1)/*n*^·kg^−1^·L^1/*n*^) is the Freundlich constant, and 1/*n* is the heterogeneity factor representing the nonlinearity of the isotherm. The estimates of the parameters and the correlation coefficient are reported in Table 6.

The Freundlich model is considered particularly suitable for heterogeneous surfaces [35]. This may reflect the heterogeneity of the adsorbent, which contains chitosan together with a ferritic core, with adsorption sites that are not all equivalent in terms of energy or affinity for the SMX. The suitability of the Freundlich model may also reflect the idea that the adsorption process is not limited to a monolayer, and multiple layers may form. This is also confirmed by the value of n > 1, which typically suggests a mechanism not strictly following ideal monolayer adsorption.

### 3.5. Comparison with Previous Studies

The results achieved within this research have been compared with those pertaining to other recent studies on SMX adsorption, as shown in Table 7. The adsorption capacity observed in this study are of the same order of magnitude for the same concentration values adopted.

## 4. Conclusions

The magnetic chitosan developed by in situ co-precipitation at a low temperature and under normal atmosphere has been successfully tested as an adsorbent to remove sulfamethoxazole, an antibiotic belonging to the sulfonamide class, commonly used to treat bacterial infections, in the presence of tertiary wastewater.

The effect of pH on SMX removal has been discussed while taking into account the charge properties of both the antibiotic and the magnetic chitosan adsorbent. The significantly different results obtained carrying out the experiments in the presence and in absence of wastewater have been discussed.

Suitable models have been selected to describe the experimental results: the Elovich model for the kinetics, and the Freundlich model for the equilibrium. These models help in understanding the mechanisms underlying the adsorption process. In particular, both models suggest an adsorption mechanism based on strong chemical interactions between the SMX and the magnetic chitosan and the formation of a multilayer adsorbate.

## Figures and Tables

**Table 1:** Physico-chemical parameters of the tertiary wastewaters.

Parameter	Value	Units
**TSS** (Total suspended solids)	2.05	mg/L
**Turbidity**	1.1	NTU
**pH**	8.7	/
**E.C.** (Electrical conductivity)	934	μS/cm
**COD_Tot_** (Total chemical oxygen demand)	26	mg/L
**COD_sol_** (Soluble chemical oxygen demand)	24	mg/L
**BOD5** (Biochemical oxygen demand)	10.3	mg/L
**Cl._Free_** (Free chloride)	0.1	mg/L
**Cl._Tot_** (Total chloride)	1.1	mg/L
**TN** (Total nitrogen)	8.92	mg/L
**N-NH_3_** (Ammonia)	0.1	mg N/L (<DL)
**N-NO_2_** (Nitrite)	0.05	mg N/L
**N-NO_3_** (Nitrate)	8.06	mg N/L
**TP** (Total phosphorus)	3.65	mg/L
**P-PO_4_** (Phosphate)	3.31	mg/L
**T** (Water temperature)	19.65	°C

**Table 2:** Main pharmaceutical pollutants contained in the tertiary wastewaters.

Compound	Class	Formula	Concentration
Clarithromycin	Antibiotic	C_38_H_69_NO_13_	<LOQ
Sulfamethoxazole	Antibiotic	C_10_H_11_N_3_O_3_S	<LOQ
Trimethoprim	Antibiotic	C_14_H_18_N_4_O_3_	<LOQ
Ketoprofen	Anti-inflammatory	C_16_H_14_O_3_	<LOQ
Carbamazepine	Antiepileptic	C_15_H_12_N_2_O	0.5
Diclofenac	Anti-inflammatory	C_14_H_11_Cl_2_NO_2_	1.2
Triclosan	Disinfectant	C_12_H_7_Cl_3_O_2_	<LOQ
Metoprolol	Betablocker	C_15_H_25_NO_3_	<LOQ
Gemfibrozil	Hypolipidemic	C_15_H_22_O_3_	<LOQ
Fluconazole	Antifungal	C_13_H_12_F_2_N_6_O	0.3
Climbazole	Antifungal	C_15_H_17_ClN_2_O_2_	0.1
Naproxen	Anti-inflammatory	C_14_H_14_O_3_	<LOQ
Flecainide	Antiarrhythmic	C_17_H_20_F_6_N_2_O_3_	0.7
Gabapentin	Antiepileptic	C_9_H_17_NO_2_	0.3
Olmesartan	Antihypertensive	C_24_H_26_N_6_O_3_	2.1
Sitagliptin	Antidiabetic	C_16_H_15_F_6_N_5_O	0.5
Telmisartan	Antihypertensive	C_33_H_30_N_4_O_2_	2.1
Venlafaxine	Antidepressant	C_17_H_27_NO_2_	0.3
Valsartan	Antihypertensive	C_24_H_29_N_5_O_3_	0.1

LOQ = Limit of quantification.

**Table 3:** Comparison between chemical composition data of the magnetic chitosan before and after adsorption of SMX solution at 50 μmol/L initial concentration, pH 3.0 for 24 h at 25 °C, obtained through EDS (results are reported as mean ± standard deviation).

Element (wt%)	C	O	Fe	N
**Magnetic chitosan**	41.1 ± 0.5	29.0 ± 0.4	20.6 ± 0.3	9.2 ± 0.8
**Magnetic chitosan after adsorption**	44.1 ± 0.4	24.0 ± 0.3	22.2 ± 0.3	9.7 ± 0.8

**Table 4:** Morphological and magnetic characteristics of the magnetic chitosan.

Reference	Particle Size (nm)	Specific Surface (m^2^/g)	Pore-Size Range (nm)	Magnetic Saturation (emu/g)
This study	16	51.3	7.23–18.0	36.6
[23]	-	47.6	12.6–49.8	43
[24]	10	101.2	4–15	-
[25]	8–10	7.31	3–27	34
[26]	9–10	68	2–50	49
[27]	-	-	-	37

**Table 5:** Estimates of the parameters contained in the Elovich model (1) and coefficient of correlation.

Parameter	C_o_ = 25 μmol/L	C_o_ = 50 μmol/L	Units
*α*	2.51 × 10^7^	2.30 × 10^4^	μmol · kg^−1^ · min^−1^
*β*	7.08 × 10^−5^	2.28 × 10^−5^	kg · μmol^−1^
R^2^	0.990	0.992	-

**Table 6:** Estimates of the parameters contained in the Freundlich model (2) and coefficient of correlation.

Parameter	Value	Units
*K_F_*	5.98 · 10^4^	μmol^(*n*−1)/*n*^ · kg^−1^ · L^1/*n*^
*n*	1.96	-
R^2^	0.997	-

**Table 7:** Adsorption capacity of SMX using different adsorbents.

Adsorbent	Reference	SMX Concentration Range, μmol/L	Maximum Adsorption Capacity, μmol/kg
Magnetic chitosan	This study	0–200	800,000
Soils	[36]	0–100	7000
Mesoporous carbon	[37]	0–200	1,380,000
Humic acid	[38]	0–400	63,200
Biochar	[39]	0–1180	5,900,000

## Data Availability

The data presented in this study are available upon request from the corresponding authors.

## Supplementary Materials

The following supporting information can be downloaded at: https://www.mdpi.com/article/10.3390/nano14050406/s1, Figures S1–S5, Tables S1 and S2, Paragraphs S1 and S2.
