# Supplementary material for: Magnetic Chitosan for the Removal of Sulfamethoxazole from Tertiary Wastewaters
Source: Nanomaterials (Basel). 2024 Feb 23;14(5):406. doi: 10.3390/nano14050406 (PMC10933842; doi:10.3390/nano14050406)
Supplement: Supplementary file 1 [file nanomaterials-14-00406-s001.zip › nanomaterials-2842938-supplementary.pdf]

nanomaterials

Supplementary materials

Magnetic Chitosan for the Removal of Sulfamethoxazole from Tertiary Wastewaters

Domenico Pirozzi [1], Alessandro Latte [1], Abu Yousuf [2], Francesco De Mastro [3], Gennaro Brunetti [3], Andrea EL Hassanin [1] and Filomena Sannino [4,*]

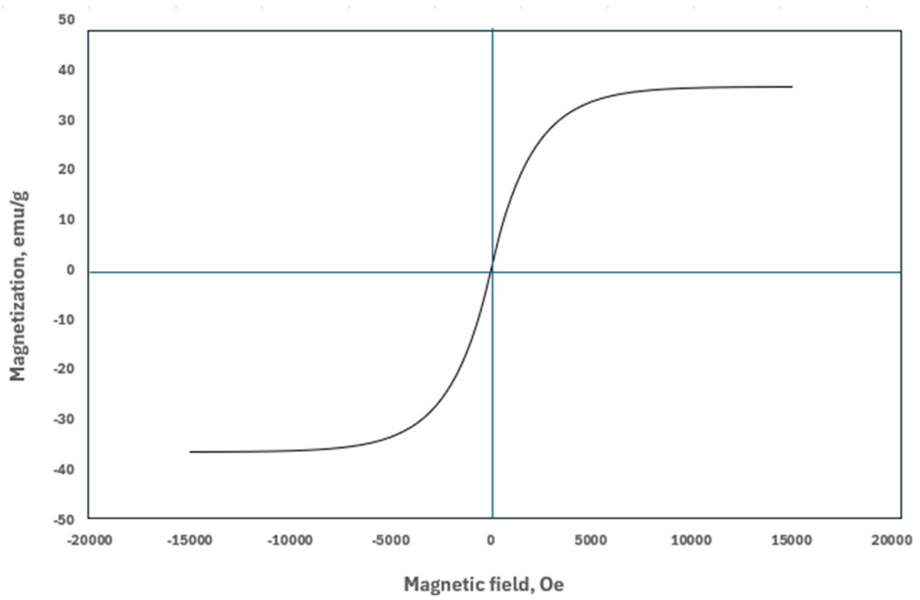

Figure S1. Magnetization curve of the magnetic chitosan.

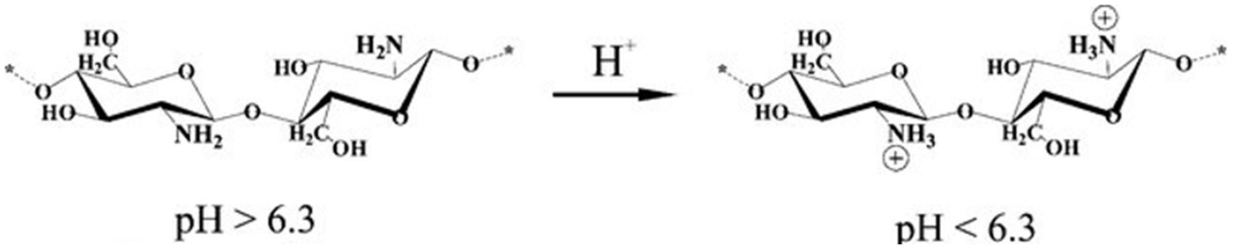

Figure S2. - Structures of chitosan at various pH values in aqueous solution. These structures contain only deacetylated glucosamine residues (from: Shchipunov et al., 2010, Green Chem., 12(7), 1187-1195).

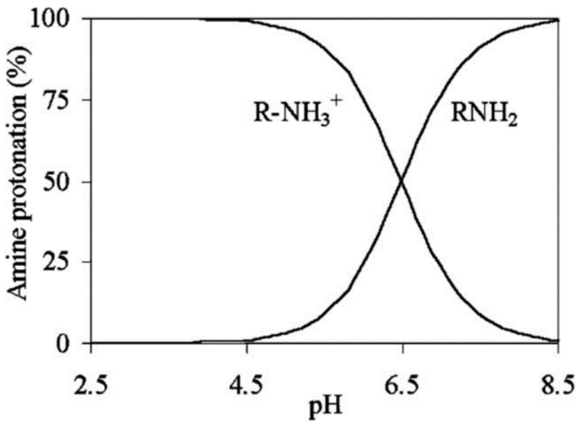

Figure S3. - Speciation of amine group as a function of pH (from: Navarro et al., 2019, Macromol. Biosci., 3: 552-561).

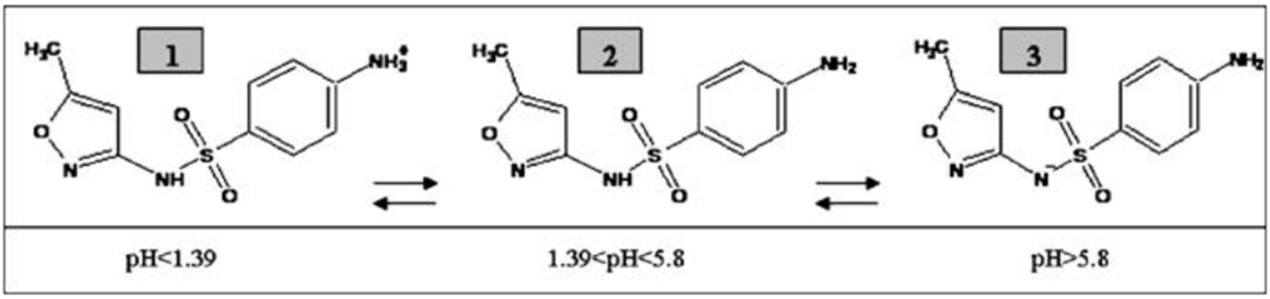

Figure S4. - Structures of SMX at various pH values in aqueous solution (form: Avisar et al., 2010, Water Air Soil Pollut., 209, 439–450).

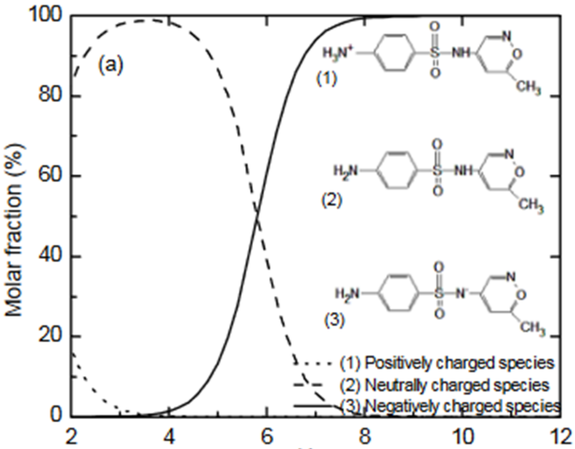

Figure S5. - Speciation of SMX as a function of pH (from: Simon et al., 2011, J. Zhejiang Univ. Sci. A **12**, 575–582).

S1. Kinetic models

Four kinetic models were taken into account describe the adsorption ofSMX on magnetic chitosan:

- pseudo-first-order (PFO)

$$\frac{dq_t}{dt} = k_{p1}(q_e - q_t)$$

where q_e and q_t are the amount of dye adsorbed (μmol·kg^{-1}) at equilibrium and at time t, respectively, k_{p1} is the rate constant of adsorption (h^{-1}) and t is the time (h). The linear form of the PFO model can be written as:

$$\log(q_e - q_t) = \log q_e - \frac{k_{p1}}{2.303}t$$

- pseudo-second-order (PSO)

$$\frac{dq_t}{dt} = k_{p2}(q_e - q_t)^2$$

-

where k_{p2} is the rate constant of adsorption (kg μmol^{-1} h^{-1}) and t is the time (min). The linear form of the PFO model can be written as:

$$\frac{t}{q_t} = \frac{1}{V_0} + \frac{1}{q_e}t$$

where:

$$V_0 = k_{p2}q_e^2$$

- intraparticle diffusion

The linear form of the intraparticle diffusion model can be written as:

$$q_t = K_{diff} \cdot t^{\frac{1}{2}} + c$$

where K_{diff} is the rate constant of adsorption (μmol·kg^{-1} h$^{-1/2}$) and c is amount of dye adsorbed (mg g^{-1}) at t=0.

- Elovich model

$$q_t = \frac{1}{\beta} ln(1 + \alpha \cdot \beta \cdot t)$$

where q_e (μmol·kg^{-1}) is the amount of SMX adsorbed per unit mass of adsorbent (adsorption capacity), α (μmol·kg^{-1}·min^{-1}) is the Elovich initial adsorption rate, β (kg·μmol^{-1}) is the desorption constant and t (min) is the time.

The estimates of the parameters and the correlation coefficient are reported in the Table S1.

Table S1. - Fitting parameters for the kinetic models for the SMX adsorption on the magnetic chitosan.

	I order			II order		
	q_e (μmol·kg^{-1})	K_{p1} (h^{-1})	R^2	q_e (μmol·kg^{-1})	K_{p2} (kg μmol^{-1} h^{-1})	R^2
**C_o = 25 μmol/L**	1.81 10^5	0.101	0.918	1.83 10^5	1.04 10^{-6}	0.966
**C_o = 50 μmol/L**	2.81 10^5	0.020	0.947	2.99 10^5	7.4 10^{-8}	0.980
	**Intraparticle diffusion**			**Elovich**		
	K_{diff} (μmol·kg^{-1} h$^{-1/2}$)	c (μmol·kg^{-1})	R^2	α (μmol·kg^{-1}·min^{-1})	β (kg·μmol^{-1})	R^2
**C_o = 25 μmol/L**	3.77 10^3	1.01 10^5	0.978	2.51·10^7	2.30·10^4	0.990
**C_o = 50 μmol/L**	6.70 10^3	9.21 10^4	0.985	7.08·10^{-5}	2.28·10^{-5}	0.992

S2. Isotherm models

Two models were taken into account describe the adsorption isotherms of SMX on magnetic chitosan:

- Freundlich model

$$q_e = K_F \cdot C_e^{1/n}$$

where q_e (μmol·kg^{-1}) is the amount of SMX adsorbed per unit mass of adsorbent (adsorption capacity), C_e (μmol·L^{-1}) is the equilibrium concentration of SMX in the solution, K_F (μmol$^{(n-1)/n}$·kg^{-1} ·L$^{1/n}$) is the Freundlich constant and $1/n$ is the heterogeneity factor representing the nonlinearity of the isotherm.

- Langmuir model

$$q_e = q_\infty \frac{KC_{A,e}}{1+KC_{A,e}}$$

where q_e is the adsorption density at equilibrium (μmol·kg^{-1}), q_∞ is the adsorption capacity of the adsorbent or the maximum achievable adsorption density (μmol·kg^{-1}), $C_{A,e}$ is the equilibrium adsorbate concentration in the liquid phase (kg/m^3), and K is the Langmuir equilibrium constant (m^3/kg).

The estimates of the parameters and the correlation coefficient are reported in the Table S2.

Table S2. - Fitting parameters for the isotherm models for the SMX adsorption on the magnetic chitosan.

Freundlich			Langmuir		
**K_f (μmol$^{(n-1)/n}$·kg^{-1} ·L$^{1/n}$)**	**n (-)**	**R^2**	**q_e (μmol·kg^{-1})**	**K** (m^3/kg)	**R^2**
5.98·10^4	1.96	0.9978	1.07·10^6	1.64·10^{-2}	0.983
